# LncRNA XIST accelerates burn wound healing by promoting M2 macrophage polarization through targeting IL-33 via miR-19b

**DOI:** 10.1038/s41420-022-00990-x

**Published:** 2022-04-21

**Authors:** Li Pi, Bairong Fang, Xianxi Meng, Li Qian

**Affiliations:** The Department of Burn and Plastic Surgery, the Second Xiangya Hospital, Central South University, Changsha, 410011 Hunan China

**Keywords:** Cell death and immune response, Trauma

## Abstract

Burn injuries are a serious threat to quality of life. The aim of this study was to investigate the mechanism of burn wound healing. The lncRNA XIST has been associated with burn wound healing, but the mechanism is not clear. In the present study, in vitro and in vivo models of burn injuries were established by thermal injury treatment of human skin fibroblasts (HSFs) and mice, respectively. Pathological changes in skin tissues were detected by haematoxylin and eosin (HE) staining. Immunofluorescence double staining was performed to detect M2 macrophages. Furthermore, the changes of cell proliferation, apoptosis and migration by CCK-8, flow cytometry, scratch and Transwell assays to evaluate the effect of XIST on burn wound healing. The binding relationships among XIST, miR-19b and IL-33 were analyzed by RNA immunoprecipitation (RIP) and dual luciferase reporter assays. Our results found that there were targeted binding sites between XIST and miR-19b, miR-19b and IL-33. We investigated whether XIST enhanced the polarization of M2 macrophages to promote the healing of burn wounds. After fibroblast burn injury, the expression levels of XIST and IL-33 increased in a time-dependent manner, whereas miR-19b expression decreased in a time-dependent manner. XIST contributed to the proliferation and migration of skin fibroblasts by inhibiting miR-19b and enhanced fibroblast extracellular matrix production by promoting the transformation of macrophages to the M2 phenotype. In short, these findings indicate that XIST can promote burn wound healing and enhance the polarization of M2 macrophages by targeting the IL-33/miR-19b axis, which may serve as a potential theoretical basis for the treatment of burn wound healing.

## Introduction

The skin is the external barrier of the human body and has rich sensory nerve endings that can sense a variety of external physical and chemical stimuli. Structurally, the skin is divided into two parts: epidermis and dermis [1]. In severe burn wounds, all layers of the skin are severely damaged, and subcutaneous tissues, blood vessels, and nerves, and skin function worsen [2]. Burn treatment is based on the depth of the injury. The regeneration of damaged skin tissue is a highly programmed process that depends on a well-orchestrated series of cellular and molecular pathways, hemostasis, inflammation, proliferation, and remodeling phases [3–5]. In the process of the wound immune response, macrophages are considered key factors that promote the inflammatory-proliferation phase transition and are divided into two main subtypes based on the activation of specific environmental signals: classically activated M1 macrophages, which promote inflammation, and activated M2 macrophages, which eliminate inflammation and repair wounds [6–8].

A variety of mechanisms of skin burn healing have been identified, including X-inactive-specific transcript (XIST). Previous studies have shown that XIST is involved in the occurrence and processes of various malignant tumors, including breast cancer, gastric cancer, hepatocellular carcinoma, and non-small-cell lung cancer, and plays oncogenic roles to promote the growth and invasion of cancer cells [9–12]. XIST has been reported to promote the repair of denatured dermis after skin thermal injury, and XIST contributes to the synthesis, proliferation and migration of the extracellular matrix of human skin fibroblasts (HSFs) by targeting miR-29b-3p/COL1A1 [13]. XIST can also inhibit miR-29a and promote LIN28A expression, suggesting a promising strategy for the repair of denatured dermis [14]. Relationships between XIST and interleukins have also been observed, usually through association with microRNAs. Analyses of abnormal microRNA expression in osteoarthritis (OA) have proved that miR-142-5p is downregulated in IL-1β-treated chondrocytes, whereas XIST levels are increased [15]. XIST binds miR-675-3p, and studies of OA development and progression have shown that miR-675-3p has antiapoptotic and chondroprotective effects in IL-1β-stimulated human chondrocytes [16]. XIST can alleviate pain behavior by suppressing inflammatory cytokine levels of IL-6 [17]. However, the exact role and mechanism of XIST in skin wound healing remain to be explored.

As an immune cytokine, interleukin-33 (IL-33) participates in the pathological process of many diseases. IL-33/innate lymphoid cell signaling is an important link between the skin epidermis and immune system and promotes the repair of skin damage [18]. However, the mechanism of IL-33 in the healing of skin injury is still unclear. Bioinformatics analyses have revealed that miR-19b can bind XIST and the 3’-UTR of IL-33. Studies have demonstrated that miR-19b increases the proliferation and migration of cardiac fibroblasts [19, 20] and inhibits the apoptosis of endothelial cells [21]. In renal injury, exosomal miR-19b acts as a mediator between tubular epithelial cells and macrophages, enhancing the activation of M1 macrophages [22]. We hypothesized that XIST promotes IL-33 by targeting miR-19b and regulates the polarization of M2 macrophages to promote burn wound healing. Our study results may provide a potential theoretical basis for the treatment of burn wound healing.

## Results

### XIST, miR-19b and IL-33 expression in denatured dermis of burn injury skin

**Fig. 1:**
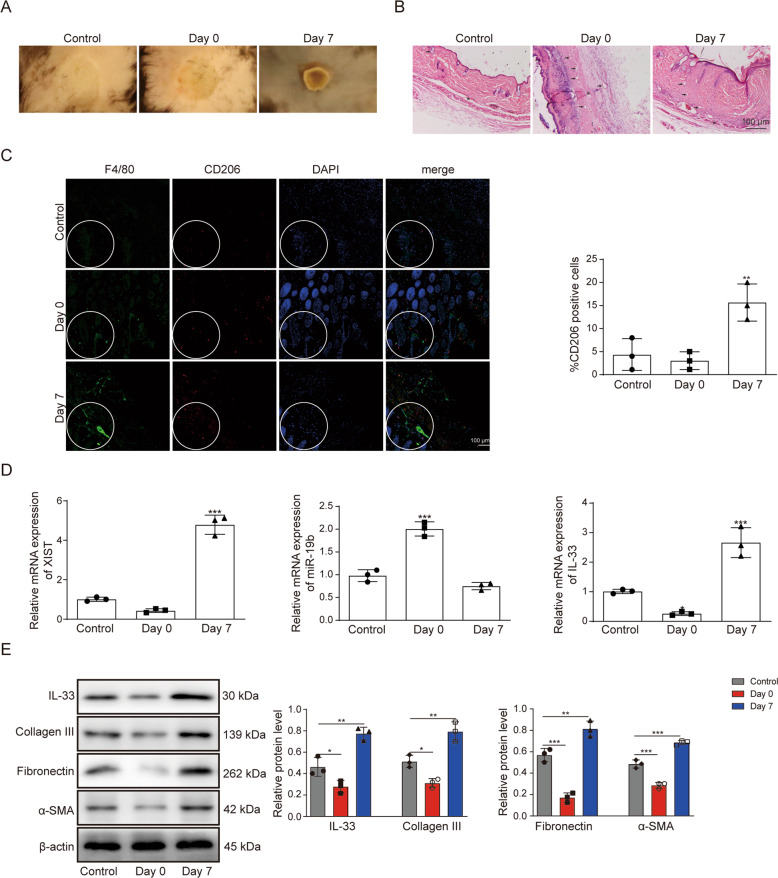
The expression of XIST, miR-19b and IL-33 in the denatured dermis of thermally injured skin. At 0 or 7 days after burn injury, **A** the skin wounds in mice were imaged. **B** The burn injury wounds of mice were analyzed by HE staining. **C** The expression of CD206 and F4/80 in mouse tissues was detected by immunofluorescence analysis. **D** RT‑qPCR detected the expression of XIST, miR-19b and IL-33 in denatured dermis. **E** Western blotting showed expression of IL-33 and extracellular matrix (ECM)-related proteins (collagen III, α-SMA and fibronectin) in the denatured dermis of mice after scalding was investigated by western blot.

In photos of the wound in the burn injury mouse models, the middle part of the wound was light red, and the periphery was light white and slightly raised, while the color of normal skin was uniform, with a flat surface. Seven days later, the wound scabbed (Fig. 1A). Histologically, compared with normal skin, part of the epidermis of the scalded skin was peeled off, many vacuoles were formed in the dermis, many voids were generated between the collagen fiber and the appendages, some of the appendages became rounded and contracted, and the connection of the muscle layer was loose. Seven days after scalding, epidermal cells and dermal cells increased significantly, voids labeled with arrows, and the connection of the muscle layer labeled with asterisks (Fig. 1B). Immunofluorescent CD206 and F4/80 double staining showed that F4/80- and CD206-positive (F4/80 + , CD206 + ) macrophages were significantly increased (Fig. 1C). The expression of XIST, miR-19b and IL-33 in denatured dermis was detected by RT‑qPCR. As shown in Fig. 1D, after scalding, the expression of XIST and IL-33 increased, while the expression of miR-19b decreased. The expression of IL-33 and the extracellular matrix (ECM) markers collagen III, α-SMA and fibronectin were increased 7 days after scalding as evaluated by western blotting (Fig. 1E). In summary, XIST and IL-33 expression increased while miR-19b expression decreased in denatured dermis after thermal injury.

### XIST and IL-33 expression is increased while miR-19b expression is decreased in thermally injured HSFs

**Fig. 2:**
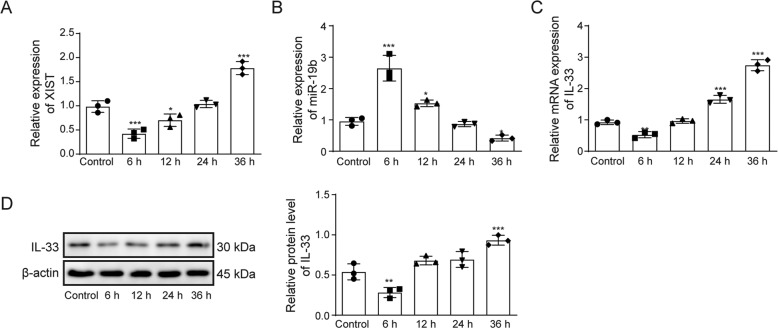
The expression levels of XIST and IL-33 were increased in thermally injured HSFs, while miR-19b was downregulated. HSFs were treated with 52 °C water for 30 s to induce thermal injury. After 6, 12, 24, 24 or 36 h of incubation, **A** the expression of XIST in HSFs was detected by RT–qPCR. **B** The expression of miR-19b in HSFs was detected by RT–qPCR. **C** The expression of IL-33 in HSFs was detected by RT–qPCR. **D** The expression of IL-33 in HSFs was detected by western blot.

To further confirm the expression levels of XIST, IL-33 and miR-19b after thermal injury, the expression levels of these proteins were evaluated in a thermal injury model of HSFs at the indicated times (6, 12, 24, or 36 h). As shown in Fig. 2A–C, RT-qPCR analysis indicated that XIST and IL-33 expression increased while miR-19b expression decreased in a time-dependent manner. Western blotting also showed that the expression levels of IL-33 increased in a time-dependent manner (Fig. 2D) and that XIST and IL-33 expression were upregulated in a time-dependent manner, while miR-19b was downregulated in a time-dependent manner.

### XIST upregulates the expression of IL-33 by binding to miR-19b

**Fig. 3:**
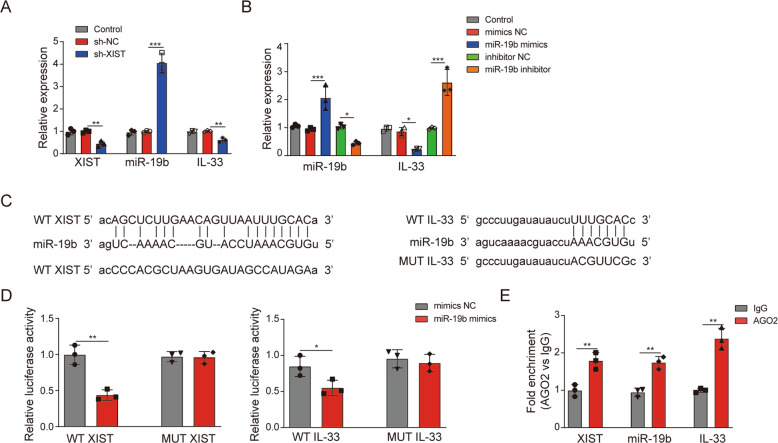
XIST upregulates the expression of IL-33 by binding to miR-19b. **A** HSFs were transfected with sh-NC or sh-XIST for 48 h. The expression of XIST, miR-19b and IL-33 in HSFs after XIST knockdown was assessed by RT–qPCR. **B** HSFs were transfected with mimics-NC, miR-19b mimics, inhibitor-NC or miR-19b inhibitor for 48 h. The expression of miR-19b and IL-33 in HSFs after miR-19b overexpression or knockdown was detected by RT–qPCR. **C** According to the starBase database, XIST has binding sites in the miR-19b gene, and IL-33 has binding sites for miR-19b. **D** HSFs were transfected with mimics-NC or miR-19b mimics. A dual-luciferase reporter assay was performed to detect the binding relationship of XIST with miR-19b and miR-19b with IL-33. **E** The RIP method was used to evaluate the binding relationship between miR-19b and IL-33/XIST.

To explore the interaction among XIST, miR-19 and IL-33, XIST was knocked down, and miR-19b mimics and miR-19b inhibitor were transfected. miR-19b and IL-33 expression increased and decreased, respectively, in response to XIST knockdown (Fig. 3A). RT‑qPCR detection confirmed that the expression of IL-33 decreased in response to transfection of HSFs with miR-19b mimics, whereas IL-33 expression increased in response to miR-19b inhibitor transfection (Fig. 3B). Bioinformatics analysis predicted that miR-19b binds XIST and the 3’-UTR of IL-33 (Fig. 3C). Furthermore, dual-luciferase reporter assays showed that XIST bound to miR-19b and that miR-19b bound to IL-33 (Fig. 3D). RIP experiments also confirmed targeted regulatory relationships between XIST and miR-19b and between miR-19b and IL-33 (Fig. 3E). In conclusion, XIST binds miR-19b, miR-19b binds IL-33, and XIST regulates IL-33 by acting on miR-19b.

### XIST promotes the proliferation and migration of HSFs by inhibiting miR-19b

**Fig. 4:**
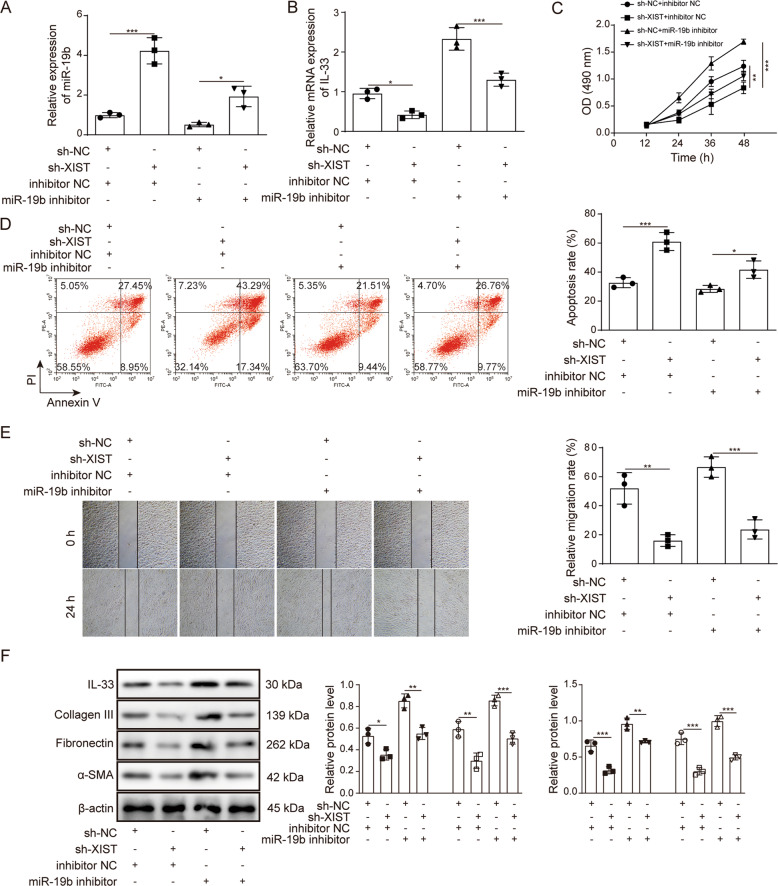
XIST promotes the proliferation and migration of HSFs by inhibiting miR-19b. HSFs were transfected with sh-NC + inhibitor-NC, sh-XIST + inhibitor-NC, sh-NC + miR-19b inhibitor or sh-XIST + miR-19b inhibitor for 48 h. **A**, **B** The expression of miR-19b and IL-33 in HSFs was detected by RT–qPCR. **C** The CCK-8 method was performed to evaluate the viability of HSFs. **D** The apoptosis of HSFs was detected by flow cytometry. **E** The migration of HSFs was assessed by a wound healing assay. **F** The expression of IL-33 and ECM markers (collagen III, α-SMA and fibronectin) in HSFs was detected by western blotting.

To further explore the effect of XIST on miR-19b, XIST was knocked down simultaneously with transfection of miR-19b inhibitor in HSFs after thermal injury. RT–qPCR detection showed that miR-19b was increased in response to XIST knockdown but decreased in response to miR-19b inhibitor transfection (Fig. 4A). However, in contrast to miR-19b, the expression of IL-33 was decreased in response to XIST knockdown but increased in response to miR-19b inhibitor transfection (Fig. 4B). In terms of cell proliferation, migration and apoptosis, when XIST was knocked down, cell proliferation decreased, apoptosis increased, and cell migration ability decreased, and these effects were reversed by miR-19b inhibitor transfection (Fig. 4C, D, E). The expression levels of IL-33 and the ECM markers collagen III, α-SMA and fibronectin were all decreased in response to XIST knockdown, and XIST inhibition was also reversed after transfection with the miR-19b inhibitor (Fig. 4F). These findings suggest that XIST inhibits miR-19b and increases the proliferation and migration of HSFs.

### XIST facilitates macrophage transformation to the M2 phenotype

**Fig. 5:**
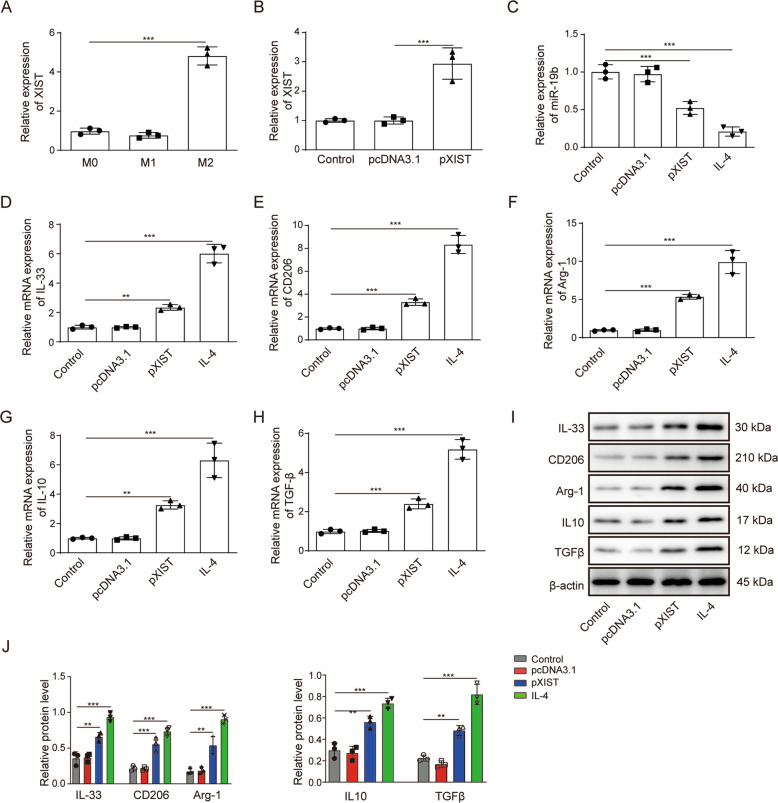
XIST facilitates macrophage transformation to M2 macrophages. **A** The expression of XIST in M0, M1 and M2 macrophages was detected by RT–qPCR. **B** M0 macrophages were transfected with pcDNA3.1 or pcDNA3.1-XIST for 48 h. The expression of XIST in M0 macrophages was measured by RT–qPCR. **C** M0 macrophages were treated with pcDNA3.1, pcDNA3.1-XIST or 20 ng/ml IL-4 for 48 h. The expression of miR-19b in M0 macrophages was analyzed by RT–qPCR after XIST overexpression. **D** M0 macrophages were treated with pcDNA3.1, pcDNA3.1-XIST or 20 ng/ml IL-4 for 48 h. The expression of IL-33 in M0 macrophages was measured by RT–qPCR. **E**–**H** M0 macrophages were treated with pcDNA3.1, pcDNA3.1-XIST or 20 ng/ml IL-4 for 48 h. The expression of M2 macrophage markers (CD206, Arg-1, IL-10 and TGF-β) in M0 macrophages overexpressing XIST was detected by RT–qPCR. **I**–**J** The expression of IL-33 and M2 macrophage markers (CD206, Arg-1, IL-10 and TGF-β) in M0 macrophages was also detected by western blotting.

To explore the regulatory effect of XIST on macrophages, macrophages were first stimulated to different phenotypes (M1 or M2). RT–qPCR detection showed that the expression of XIST was significantly higher in M2 macrophages than in M1 macrophages (Fig. 5A). Transfection of IL-4-treated M0 macrophages with the XIST overexpression construct significantly increased the expression of XIST (Fig. 5B) and decreased the expression of miR-19b (Fig. 5C). The expression of IL-33 was increased in response to overexpression of XIST and significantly increased in IL-4-treated M0 macrophages with a consistent trend (Fig. 5D). Overexpression of XIST resulted in significantly increased expression of the M2 macrophage markers CD206, Arg-1, IL-10 and TGF-β, suggesting that XIST promotes macrophage transformation to the M2 phenotype (Fig. 5E–H). Western blotting also showed that the expression of IL-33 and the M2 macrophage markers CD206, Arg-1, IL-10 and TGF-β were all increased in response to overexpression of XIST in M0 macrophages (Fig. 5I). To summarize, XIST facilitates the transformation of macrophages to the M2 phenotype.

### XIST contributes to fibroblast proliferation and EMC production by promoting M2 macrophage activation

**Fig. 6:**
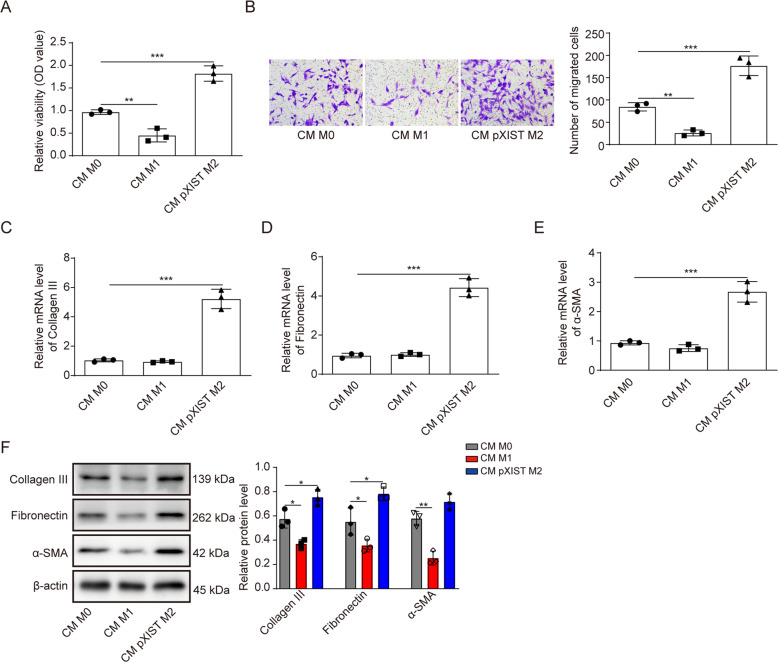
XIST contributes to fibroblast proliferation and EMC production by promoting M2 macrophage activation. HSFs were treated with culture medium from M0, M1 or M2 macrophages induced by XIST overexpression for 48 h. **A** The CCK-8 assay was performed to evaluate the proliferation of HSFs. **B** The migration of HSFs was assessed by the Transwell assay. **C**–**E** The expression of ECM markers (collagen III, α-SMA and fibronectin) in HSFs was detected by RT–qPCR. **F** The expression of ECM markers (collagen III, α-SMA and fibronectin) in HSFs was detected by western blotting.

To investigate the relationship between XIST-induced M2-type polarization of macrophages and HSF cell proliferation, a coculture model with HSF and THP-1 cells was established. THP-1 cells were induced to form M0 macrophages by PMA and then treated with IFN-γ (100 ng/mL) or IL-4 (20 ng/mL) for 48 h to induce polarization to the M1 and M2 phenotypes, respectively. The CCK-8 assay showed that the proliferation of HSFs was reduced by treatment with M1 macrophages but increased by treatment with XIST overexpression-induced M2 macrophages (Fig. 6A). The Transwell migration assay showed that the migration ability of HSFs was increased by treatment with XIST overexpression-induced M2 macrophages (Fig. 6B). RT–qPCR and western blotting were performed to further detect the mRNA levels of ECM markers in the three HSF groups. As shown in Fig. 6C–F, the expression levels of the ECM markers collagen III, α-SMA and fibronectin were increased in HSFs treated with XIST overexpression-induced M2 macrophages, suggesting that XIST enhances HSF proliferation and EMC production by promoting M2 macrophage activation.

## Discussion

There are many factors leading to skin injury, such as physical, chemical and biological injury factors. Burns are a major cause of skin injuries with serious consequences, such as death, loss of limb function, and disfigurement, and often require a long rehabilitation period. The extreme pain of skin grafting and physical therapy cause physical and psychological trauma for the victim for life. Many basic research and clinical explorations of burn wound healing have been conducted [3–5, 13, 14, 18, 23], but wound healing remains a difficult clinical problem due to the complexity of the process, which involves the participation and mutual regulation of cells and cytokines. Research on the mechanism of burn wound healing is still an urgent need to develop specific treatment methods. In this study, we investigated the role of the lncRNA XIST in burn wound healing and found that XIST binds IL-33 through miR-19b and enhances the polarization of M2 macrophages to promote burn wound healing.

In recent years, cellular and molecular pathways related to wound healing have been widely studied. Stimulation of some signaling pathways is necessary for directional wound healing. Long noncoding RNAs (lncRNAs) are gene transcripts with lengths greater than 200 nucleotides that lack the capacity for protein encoding [24]. Compared with miRNAs, there are more types of lncRNAs, and their functions and mechanisms are more complicated. LncRNAs can inhibit miRNA expression by acting as sponges or ceRNAs in many diseases [25] ([26]. Only a few lncRNA gene structures have been identified, and XIST is one of the earliest discovered lncRNA genes. XIST acts as a regulator of the occurrence and development of diseases [27–30]. XIST serves as a decoy of many miRNAs, such as miR-29b-3p, miR-106b-5p, and miR-140-5p [13, 31, 32]. Here, we found that XIST expression was increased after thermal injury, in agreement with previous studies [13, 14]. In addition, our data revealed that miR-19b binds XIST, and IL-33 was identified as the target of miR-19b. Thus, our findings indicate that XIST regulates the proliferation, migration and apoptosis of HSFs by indirectly targeting IL-33.

We also confirmed that XIST binds miR-19b and that miR-19b binds IL-33 in HSF cells by bioinformatics analysis and luciferase reporter assays. Loss-of-function experiments showed that silencing XIST decreases the proliferation and migration of HSFs and the expression of IL-33 and EMC markers. However, this effect was regulated by miR-19b. miR-19b is generally expressed in cardiomyocytes, monocytes and vascular endothelial cells and is closely associated with cardiovascular and kidney diseases. This paper is the first to reveal the mechanism of miR-19b and XIST in burn wound healing.

Interleukin-33 (IL-33), a member of the IL-1 cytokine family with pleiotropic specific immune effects, is involved in the pathological processes of allergic asthma, arthritis, type I diabetes and severe infections [33, 34]. When tissue cells are stimulated or damaged, IL-33 is released outside as an alertin, which specifically binds to the suppression of tumorigenicity 2 (ST2) receptor and activates T helper 2 (Th2) cells. Then, the cytokines IL-4 and IL-13 are expressed to regulate the function of epithelial cells and fibroblasts. IL-33 is considered to play a crucial role in the regulation of diabetic wound healing by upregulating the expression of M2 macrophages [23]. In this research, we found that XIST indirectly targets IL-33. Thus, XIST upregulation may regulate the function of HSFs by indirectly targeting IL-33 to activate Th2 cells. In future studies, we will further study the role of XIST in Th2 cell functions.

Wound healing processes require the production of many cytokines, a major source of which are macrophages [35]. A previous study examined the role of XIST in the regulation of macrophage polarization, which is positively associated with lung cancer-conditioned tumor-associated macrophages (M2 phenotype) [30]. The lncRNA GAS5 enhances the M1 macrophage phenotype in the pathogenesis of impaired diabetic wound healing [36]. There is evidence that this differential polarization of macrophages in diverse infectious disease conditions demonstrates the plasticity of these cells, with M1-like polarization evident in inflammatory diseases, whereas M2-like polarization has been proposed in chronic parasitic, viral, or bacterial diseases [37]. M1 macrophages represent a proinflammatory phenotype, with increased phagocytic capacity, antigen-presenting activity, proinflammatory cytokine secretion, and oxidative metabolites to promote host defence and elimination of damaged tissues. During the early inflammatory phase, M1 macrophages are important in the clearance of dead cells and debris within the wound [37]. In our study, we found that the significant upregulation of XIST in wounds was associated with increased expression of M2 macrophage marker RNAs (CD206, Arg-1, IL-10 and TGF-β), demonstrating for the first time that XIST contributes to HSF proliferation, migration and ECM synthesis by activating M2 macrophages. This is consistent with previous findings that IL-33 upregulates the expression of M2 macrophages in diabetic skin wounds [23]. In addition, we demonstrated that XIST promotes burn wound healing through targeting of IL-33 by miR-19b.

**Fig. 7:**
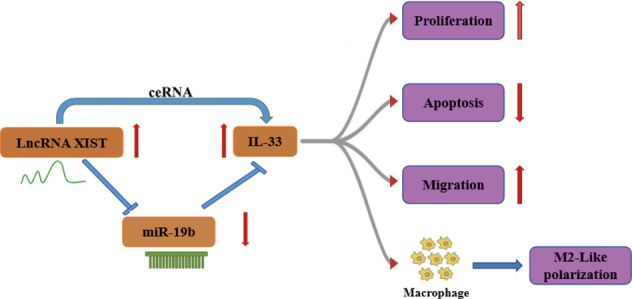
Schematic diagram of the mechanism and function of XIST. XIST acts as a ceRNA against miR-19b and upregulates IL-33 expression. IL-33 promotes proliferation, migration and M2-like polarization, thereby promoting burn wound healing.

In conclusion, the present study suggests that XIST binds to miR-19b, which targets IL-33 and activates M2 macrophages in burn wound healing (Fig. 7). The XIST/miR-19b/IL-33 axis plays a critical role in denatured dermis repair after thermal injury. However, we collected relevant data for only one week, and further clinical verification is needed. If the results are consistent with those at the cellular and animal levels, it could provide a target for the development of therapeutic drugs for the treatment of skin burn wound healing in the future.

## Materials and methods

### Animal models of burn injury

Male C57BL/6 mice (*n* = 30; age, 5-6 weeks; weight, 20 ± 2 g) were purchased from the Shanghai Laboratory Animal Center (Shanghai, China). Animals were housed in separate cages in a temperature-controlled room at 22–25 °C with 12 h of light and 12 h of darkness and had free access to water. Throughout the study, all experimental operations were approved by the Ethics Committee of Second Xiangya Hospital of Central South University. After 3 days of domestication, the mice were anaesthetized by intraperitoneal injection of 10 g/L pentobarbital sodium (Alfasan Co., Holland), and back cutaneous hair was removed. For the mice in the experimental group, the site of hair removal was exposed to water heated at 80 °C for 100 s to create a 10-mm-diameter wound. The mice in the control group received no treatment except removal of the back cutaneous hair. Mice were sacrificed for sampling on the first day and the seventh day. Photos were taken, and wound skin tissues were excised for subsequent testing.

### Haematoxylin–eosin staining

Mouse skin was stained with haematoxylin–eosin (HE) to observe changes in the burn injury wounds. Samples of the burn wound and surrounding skin with a size of approximately 0.5 cm were spread on filter paper, fixed in 10% formalin, and then cut on a microtome to widths of 3–4 mm vertically and horizontally. Paraffin sections were prepared according to the routine method into 5-μm sections. The tissues were stained using an HE staining kit (Beyotime, Shanghai, China) and then observed under an optical microscope (400×; ERc5S, Carl Zeiss Company, Oberkochen, Germany).

### Cell culture and treatment

Human skin fibroblast (HSF) cell lines were obtained from the Cell Bank of the Chinese Academy of Sciences (Shanghai, China), suspended in 10 mL of DMEM with 10% FBS and cultured in an atmosphere containing 5% CO_2_ at 37 °C. HSFs were inoculated at a cell number of 1 × 10^6^ cells into100-mm-diameter culture dishes. When the cells reached 50%~60% confluence, the cell suspension was incubated in 52 °C water for 30 s. In the control group, the suspension of HSFs was incubated in 37 °C water for 30 s. Four groups of HSFs were obtained according to different time points after thermal injury (6 h, 12 h, 24 h, 36 h).

The human monocyte leukemia cell line THP-1 was purchased from American Type Culture Collection (ATCC). The cells were cultured in RPMI-1640 (Solarbio. Co, Beijing, China) supplemented with 10% FBS (Gibco Company, USA). After 4–6 generations, THP-1 cells were seeded at a density of 3×10^6^ cells into a 6-well plate. When cell confluence reached 90%, phorbol 12-myristate 13-acetate (PMA, Beijing Noble Ryder Co. 320 nM) was added to the culture medium. After 48 h of exposure, the PMA-containing medium was replaced. THP-1 cells were differentiated into M0 macrophages. Cells were cultured with IFN-γ (100 ng/mL) and IL-4 (20 ng/mL) for another 48 h to generate M1 macrophages and M2 macrophages, respectively. The differentiated THP-1 macrophages were divided into the M0 macrophage group, M1 macrophage group and M2 macrophage group for subsequent experiments.

### Cell transfection

Short-hairpin RNA plasmid directly knocking down XIST (sh-XIST), miR-19b mimics, miR-19b inhibitor, nontargeting sequence sh-NC, mimics NC, inhibitor NC, pcDNA3.1 XIST, OE-IL-33 and empty vector were synthesized by GenePharma (Shanghai, China). Oligonucleotide or plasmid transfection into HSF cells was carried out using Lipofectamine 3000 (Invitrogen, Carlsbad, CA, USA) according to the manufacturer’s protocol. Cells were collected for further experiments at 48 h after transfection.

### Cell proliferation detection

Both HSFs and macrophages in logarithmic phase were digested and seeded into 96-well plates at 2 ×10^3^ cells per well. Then, 20 μL of 5 mg/mL CCK8 solution (Sigma, Missouri, USA) was added and incubated for 4 h at 37 °C in the dark. After discarding the culture medium, DMSO was added at 150 mL/well and mixed well to fully dissolve the CCK8 reduction product. The 96-well plate was placed in the enzyme labeling apparatus. Finally, the absorbance (OD) values of each well at a wavelength of 570 nm were measured. The OD values of each group were averaged, and the standard deviation was calculated. Each sample was repeated three times.

### Flow cytometry

Cell apoptosis was measured by flow cytometry as previously described [38]. The Annexin-VFITC/PI (propidium iodide/fluorescein isothiocyanate) Apoptosis Assay Kit (Thermo Fisher Scientific, Inc. USA) and BD FACSCalibur (BD Biosciences, San Jose, CA, USA) were used according to the manufacturer’s instructions. In brief, HSFs were resuspended in ice-cold PBS and 10% FCS and incubated with 5 μL of Annexin-V-FITC reagent and 5 μL of PI in the dark for 15 min, followed by flow cytometry analysis.

### Immunofluorescence staining

M2 macrophages were subjected to immunofluorescence double staining by performing CD206 and F4/80 overlay staining. The staining results were analyzed by two investigators. Frozen skin tissue sections were incubated with rat anti-mouse CD206 (1:100, BioLegend, San Diego, CA) and rabbit anti-mouse F4/80 primary antibodies (1:100, Abcam, Cambridge, MA) for 1 h at room temperature and then visualized with secondary antibodies (1:200, Earthox, Millbrae, CA) under a confocal microscope. The CD206 expression level was calculated by measuring the integrated optical density of images of an equivalent area by the Fluoview FV1000 Version 2.1 confocal microscope (Olympus).

### Reverse transcription‑quantitative polymerase chain reaction (RT‑qPCR)

Total RNA was extracted using TRIzol (Invitrogen; Carlsbad, CA, USA) and reverse transcribed to cDNA using a Superscript III RT kit (Invitrogen). RT–qPCR was performed using the BIO-RAD CFX96. The primers with the following sequences were synthesized: XIST Forward, 5’- CTTGGATGGGTTGCCAGCTA-3’; reverse, 5’-TCATGCCCCATCTCCACCTA-3’; miR-19b forward, 5’‑CGCTGTGCAAATCCATGCAA‑3’; reverse, 5’-GTCGTATCCAGTGCAGGGTCCGAGGTATTCGCACTGGATACGACTCAGTT‑3’; IL-33 forward, 5’‑TTATGAAGCTCCGCTCTGGC‑3’; reverse, 5’-CCAAAGGCAAAGCACTCCAC‑3’; U6 forward, 5’-CTCGCTTCGGCAGCACA-3’; reverse, 5’-AACGCTTCACGAATTTGCGT-3’; β-actin forward, 5’-CCCTGGAGAAGAGCTACGAG-3’; reverse, 5’-CGTACAGGTCTTTGCGGATG-3’. U6 and β-actin were used as internal reference genes. The 2^‑ΔΔCt^ method was used to evaluate the relative expression of the target gene.

### Western blot analysis

Cells were lysed in RIPA lysis buffer (89900, Thermo Scientific, USA) supplemented with protease and phosphatase inhibitors for 30 min. The total protein concentration was measured by a Pierce BCA Protein Assay Kit (Thermo Scientific, USA). Equal masses of proteins were resolved by sodium dodecyl sulfate-polyacrylamide gel electrophoresis and blotted onto PVDF membranes (Millipore, Bedford, USA). The membrane was blocked in 5% nonfat dry milk and incubated with the following primary antibodies for 12 h at 4 °C: anti-IL-33 (1:2000, Abcam), anti‑collagen III (1:1000, Abcam), anti‑fibronectin (1:1000, Sigma Chemical Co.), anti‑α‑SMA (1:1,000, Abcam), anti-Arg-1 (1:1000, Abcam), anti-CD63 (1:2000, Abcam), anti-IL-10 (1:2000, Abcam), anti-TGF-β (1:1000, Santa Cruz Biotechnology Inc.), or anti-β-actin (1:5000, Santa Cruz Biotechnology). The membranes were subsequently incubated with HRP‑linked goat anti-rabbit IgG secondary antibody (1:5000, ZSGB‑BIO, Beijing, China). Thereafter, an enhanced chemiluminescence kit (ECL; Millipore) was applied for the detection of immunoreactive bands.

### Wound healing assay

Cells were seeded into 24-well dishes, and when they reached 95% confluence, the monolayers were scratched with a sterile 200-μL pipette tip. Then, the cells were washed with PBS three times, and serum-free DMEM was added. The dishes were incubated at 37 °C for 24 h. Images were acquired, and the migration area of cells was measured using Photoshop software.

### RNA immunoprecipitation assay

RNA immunoprecipitation (RIP) assays were performed with the RIP kit (Cell Signaling Technology, Inc., Danvers, MA, USA) based on previously described methods [39]. The HSFs were lysed in RIP buffer for 1 h. Then, Ago2 antibody (Abcam, Cambridge, MA) and negative control normal rabbit IgG antibody (Abcam, Cambridge, MA) were incubated with the cell lysate. RNA was extracted from the immunoprecipitates and analyzed by RT–qPCR.

### Bioinformatics analysis and dual-luciferase reporter assay

Bioinformatics analysis was used to explore the binding sites of miR-19b on XIST and IL-33 by the starBase database, and the putative binding sequences were confirmed. Wild-type (WT) and mutant (MUT) 3’-untranslated region (3’-UTR) versions of XIST were inserted into the pmirGLO vector (Promega, Madison, WI, USA) to generate XIST-WT and XIST-MUT. WT and MUT 3’-UTR versions of IL-33 were cloned into the pmirGLO vector to generate IL-33-WT and IL-33-MUT. HSF cells were cotransfected with miR-19b mimics or miR-NC using Lipofectamine 2000 transfection reagent (Thermo Fisher Scientific, Inc.). The dual luciferase reporter assay was performed 48 h post transfection using the Dual Luciferase Assay System (Promega, Madison, WI, USA). All experiments were performed in triplicate.

### Statistical analysis

All experiments were performed at least three times, and SPSS software was used for statistical analysis (SPSS Inc., USA). Data are presented as the mean ± standard deviation. The unpaired two-tailed Student’s t-test was used to compare differences between two groups. One-way analysis of variance (ANOVA) followed by Tukey’s post hoc test was used for multiple comparisons. *P* < 0.05 was considered to indicate a statistically significant difference.

## Supplementary information

raw data

Language Editing Certificate

## Data Availability

All data generated or analyzed during this study are included in this published article.
