# Supplementary figures and images for: LncRNA XIST accelerates burn wound healing by promoting M2 macrophage polarization through targeting IL-33 via miR-19b
Source: Cell Death Discov. 2022 Apr 21;8:220. doi: 10.1038/s41420-022-00990-x (PMC9023461; doi:10.1038/s41420-022-00990-x)

Fig1E


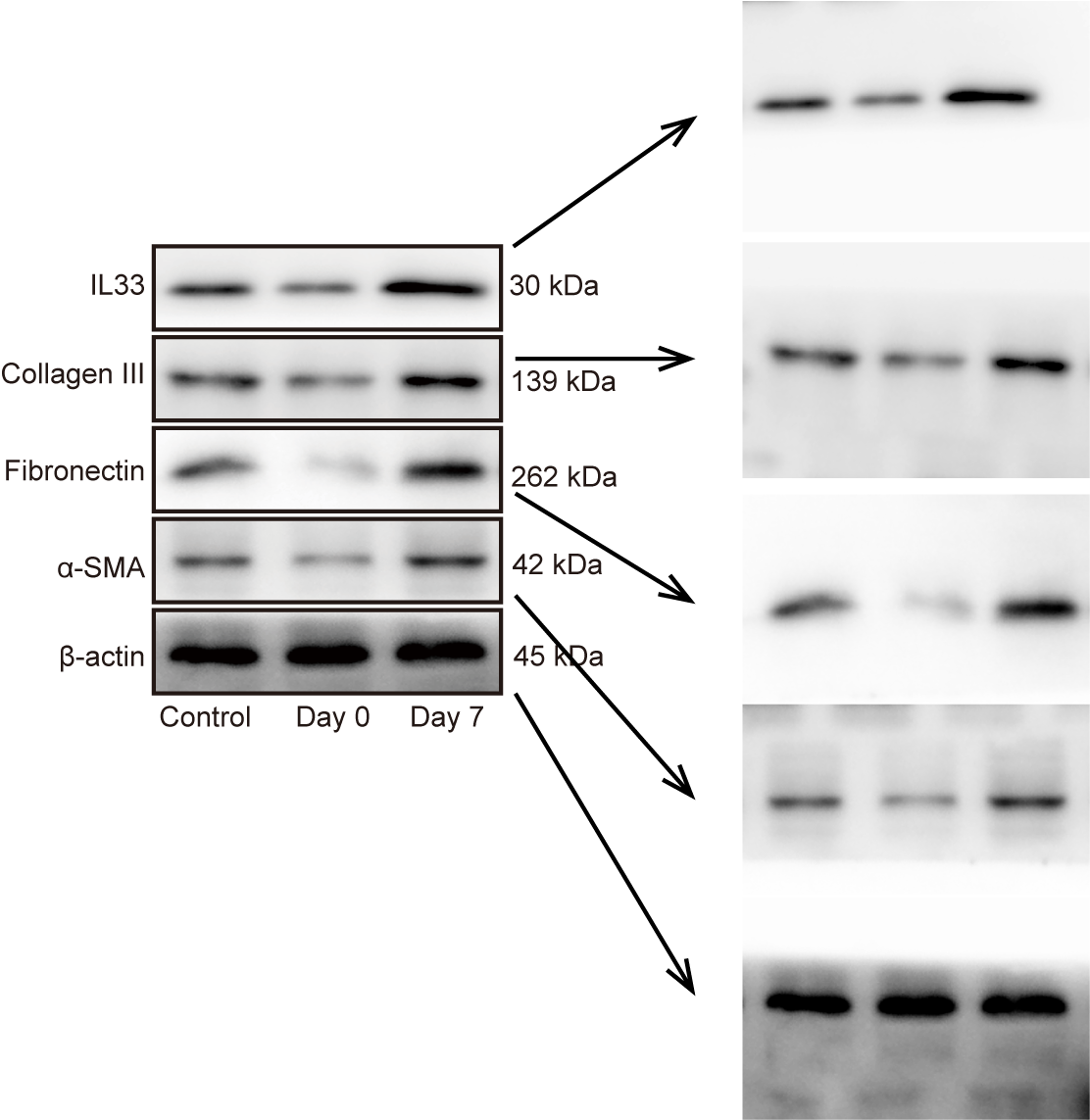


Fig2D


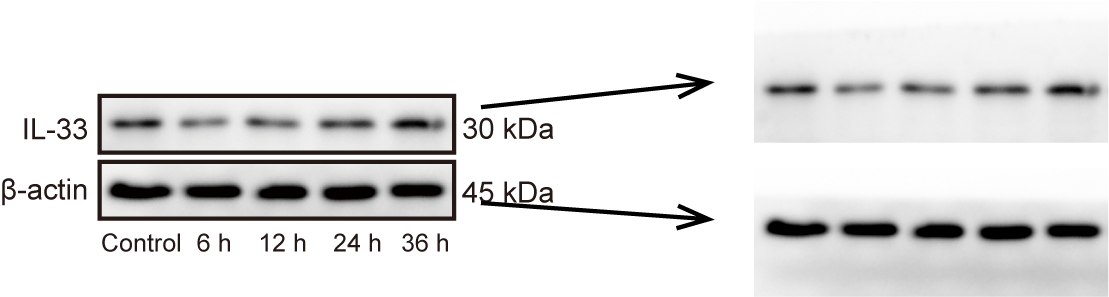


Fig4F


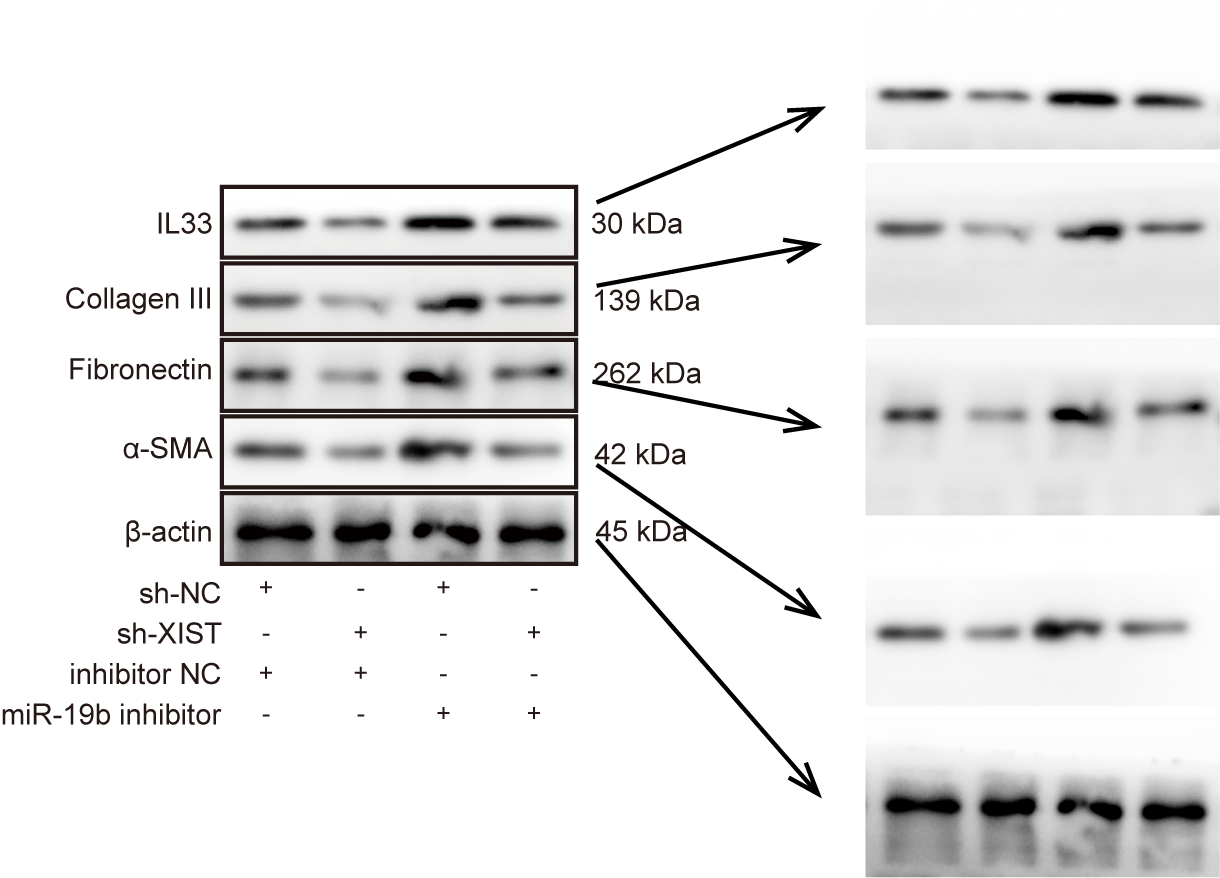


Fig5I


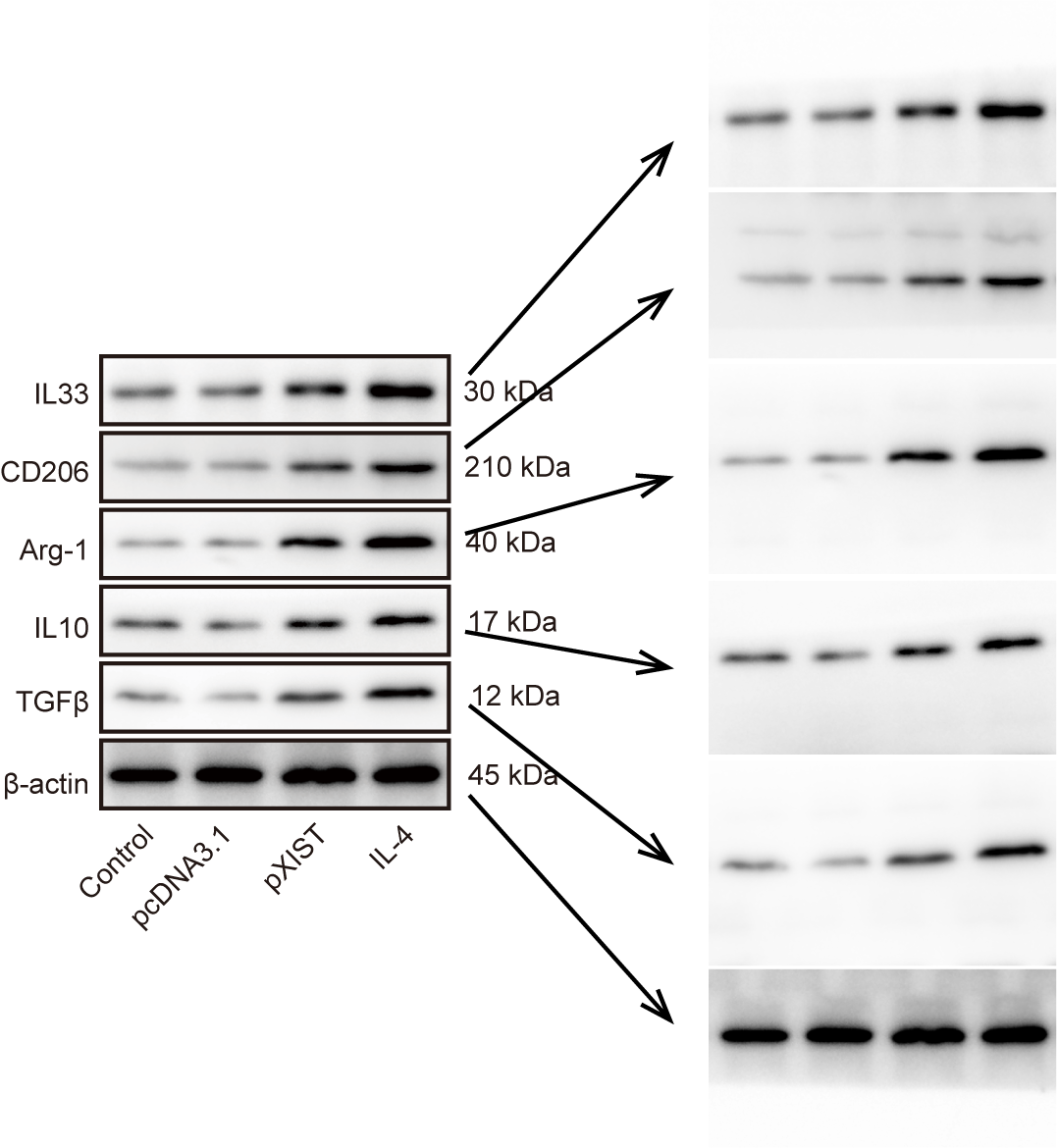


Fig6F


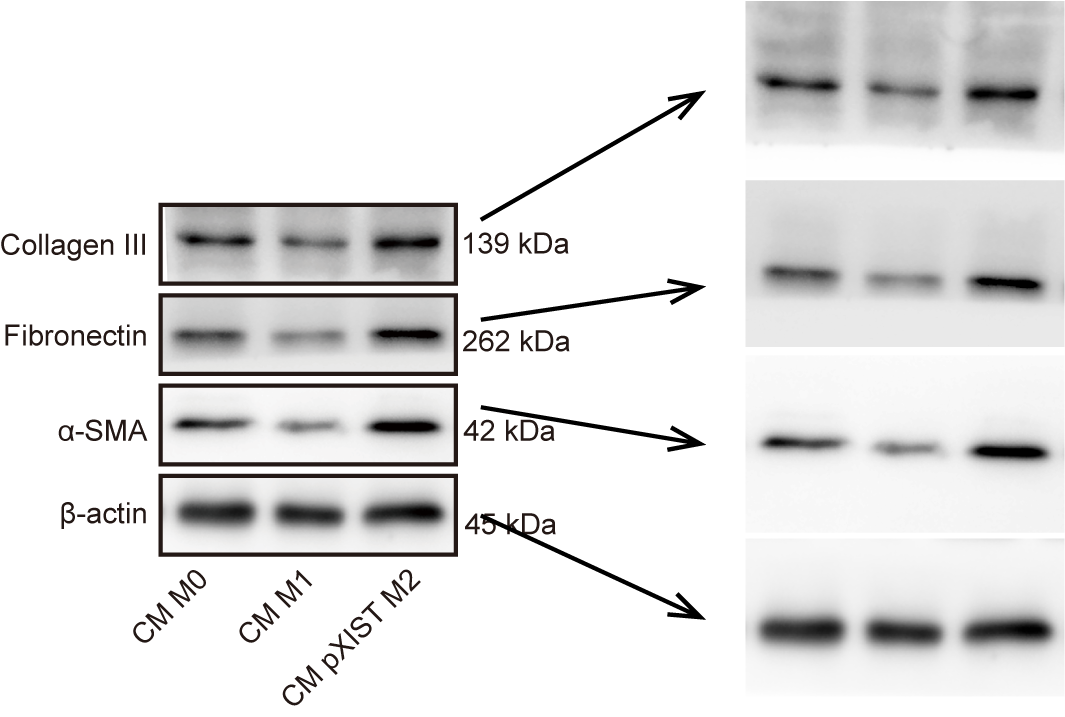

Supplement: Supplementary file 1 — raw data [file 41420_2022_990_MOESM1_ESM.docx]
